# The unwritten rules and HIV: a qualitative study of informal institutions and HIV vulnerability among workers at social venues in Uganda

**DOI:** 10.3389/fpubh.2023.1288058

**Published:** 2023-12-14

**Authors:** Susan Babirye, Kristien Michielsen, Freddie Ssengooba

**Affiliations:** ^1^International Centre for Reproductive Health, Department of Public Health and Primary Care, Faculty of Medicine and Health Sciences, Ghent University, Ghent, Belgium; ^2^Department of Health Policy, Planning, and Management, School of Public Health, College of Health Sciences, Makerere University, Kampala, Uganda; ^3^Department of Research and Evaluation, Afrislum Uganda, Kampala, Uganda; ^4^Center for Policy and Management Science, Kampala, Uganda

**Keywords:** informal institutions, workplace policies, HIV vulnerability, young people, social venues, qualitative research, Uganda

## Abstract

**Introduction:**

There is increasing appreciation of the need to understand how social and structural factors shape HIV risk. The unwritten rules, also known as informal institutions or social norms, are increasingly recognized as important determinants of HIV transmission. Unfortunately, these informal institutions, especially among high-risk environments for HIV, such as social venues like bars, lodges, remain poorly understood. This study explored the informal institutions at social venues, and how these institutions influenced vulnerability for HIV for venue workers in Uganda.

**Methods:**

We conducted a qualitative study in two districts of Kyotera and Rakai in Central Uganda. We purposively selected and interviewed 44 workers including, cleaners, waiters, and waitresses and 22 venue managers at 22 social venues to explore the informal institutions at these establishments and how the institutions shaped HIV vulnerabilities among the workers. 31.8% (14) of the participants were males, and 68.2% (30) females. Data were analyzed using thematic content analysis.

**Results:**

We found that the informal institutions at the venues were both officially and socially created, communicated, and sanctioned. The most common institutions operated through; selective hiring, rigid reporting structures, and informal job contracting procedures. Meager salaries, varying and delayed payments as well as attractive benefits and bonuses from customers were also important forms of informal institutions at the venues. Drinking alcohol, and offering sexual services at the venues were acceptable, although excessive drinking, and committed sexual relationships with customers were disapproved. These informal institutions shaped a risk environment at the venues by creating risk exposure opportunities that influenced workers' engagement into sexual risk behaviors.

**Conclusion:**

The risk environment at social venues is shaped by the informal institutions at these venues. Thus, the need for venue-based HIV programs that integrate social norms interventions to better address the contextual determinants of HIV risk behaviors at the venues.

## Introduction

Informal institutions or the unwritten rules are recognized as important determinants of HIV transmission or acquisition (1). Informal institutions are defined as “the perceived informal, mostly unwritten rules, that define acceptable and appropriate actions within a given group or community, thus guiding human behavior” (1–4). They include (1) social and organizational roles; (2) organizational hierarchies; (3) organizational culture; acceptable behavioral norms governing interactions between organizational members; and (4) shared esthetic and moral evaluations. Informal institutions are usually created, communicated, and enforced outside of officially sanctioned channels (5). Understanding the informal institutions and how they influence HIV vulnerability matters. It will inform HIV interventions that go beyond individual-level activities and spark collective-level change through addressing social structural factors that shape and determine the risks at the venue work environment. Social venues are public places—night clubs, bars, hotels, restaurants, karaoke parlors, video halls, massage parlors, and dance halls, where one can go to relax, have fun, drink alcohol or find a sexual partner. Employment linked with social venues is of great importance to the economy, particularly in terms of job creation, tax revenues and foreign currency. In the recent times, such venues have provided employment to about 144 million workers worldwide (4.6% of total global employment) (6).

Although extensive research has examined the structural factors that directly affect HIV risk and vulnerability in different high risk sub-populations (7–10), the informal institutions that underpin social interaction, culture and work setting at social venues and their implications on HIV risk remain less studied. Workers of social venues are recognized as potential high risk sub-population (11–13) with unique exposure to high risk working environments that increase their HIV burden.

In Uganda, where this study was conducted, social venues include small, medium, and big or large size venues both formal and informal, licensed, and unlicensed. Despite sex work being illegal in Uganda, the practice is evident at some venues and workers at venues with sex work, may or may not engage in sex work. The regulatory environment for social venues is still limited (14, 15) which poses a challenge for decent work as the majority of the work at social venues remain insecure, poorly paid, and often unsafe. Unfortunately, these venues are potential employment places for many young people, especially those with restrictive educational backgrounds. This is so because social venues have different roles i.e., cleaners, entertainers, bar attendants, cooks, security guards, managers, and waiters and waitresses, which require varying skills. Social venues are known “hotspots” for reaching adolescent girls and young women at risk of HIV (16), however, the informal institutions that shape the risk at the venue work environments remain poorly understood.

Whereas, there have been very few HIV epidemiological studies conducted among workers of social venues in Uganda and sub-Saharan Africa at large, a body of evidence from Asia indicates that workers of social venues engage in risk behaviors (17–20). For example, workers of social venues have been reported to engage into sex work with their patrons to supplement their income (13, 21). Studies among entertainment workers in Cambodian cities have also reported multiple concurrent sexual partners, inconsistent condom use, alcohol consumption, and sex work among workers of entertainment places. Another study highlighted that women working in entertainment services like bars have a higher HIV infection rate than the general population, which is an indication of risk sexual behaviors at worker places (19).

In Uganda, earlier research among workers of social venues reported high prevalence of risk sexual behaviors (11). These included sexual partner acquisition, multiple concurrent sexual partnerships, low condom use, and a high burden of HIV among this sub-population (venue workers). In the same study, the prevalence of HIV was highest among female workers (11%) compared to 6.3% in the general population of Uganda (22). Among young workers (15–24 years), HIV prevalence was at 6.4% with young female workers having the highest prevalence of 9%. Moreover, workers of social venues are typically young people (11, 13). High HIV prevalence rates (estimated at 19–26%) among venue workers have also been documented in a similar setting (Tanzania) (23, 24). Previous research has characterized workers of social venues as a less educated sub-group, often unmarried, and having a low income, which factors are known drivers of HIV (11, 17). Unfortunately, the already existing HIV vulnerability factors among venue workers such as age, physical, emotional, financial, and psychological dependence are intensified by the risk work environment at the venues.

Previous research that has attempted to explain the underlying reasons for the heightened practice of risk sexual behaviors, and the high burden of HIV among venue workers has focused mainly on individual level risk factors (like knowledge, attitudes, and behaviors) (11, 25–28). Thus, failing to acknowledge the social structural factors that shape and determine the working environment at the venues where risk sexual behaviors may be promoted by the “rules of the game” (29, 30). There is increasing recognition that structural factors such as informal institutions play a critical role in structuring risk environments (29, 31). They do so by (1) constraining and enabling actors, and (2) providing incentives that drive actors' decisions, preferences, and practices. Unfortunately, informal institutions at risk work environments such as social venues are not well-studied and utilized in designing HIV risk reduction interventions. Improving our understanding of the informal institutions at social venues—what they are and the dynamic between them and risk sexual behaviors could inform venue-based HIV prevention efforts in terms of rules, cultural changes to inculcate to minimize transmission risk at the venues.

In this paper, informal institutions of social venues and how they influence HIV vulnerability among venue workers (15-24) in Uganda were explored using a model of HIV risk environment by Rhodes and Simic (29). The HIV risk environment model shifts the focus from individualistic approaches to risk behaviors toward an understanding of how structural and environmental conditions shape an individual's vulnerability to HIV acquisition. Risk environment is defined as the space (physical or social) in which four types of environmental influence factors; physical, social, economic, and policy interact at both micro and macro levels to increase the chances of acquiring HIV in a given environment. The micro-risk level environment focuses on personal decisions as well as the influence of community level norms and practices. The macro-risk level environment seeks to capture structural factors, such as rules, economic conditions, and institutional settings. Rhodes and Simic's risk environment framework is useful because it highlights the multilevel and contextualized nature of HIV risk and focuses on interactions between risk factors exogenous to the individual, rather than endogenous factors, such as risk practices, age, or sex.

## Materials and methods

This exploratory qualitative study was conducted between October and December 2021 in greater Rakai district, in the central region of Uganda. We used in-depth interviews (with workers and managers) to explore the informal institutions at social venues and how the institutions shaped HIV vulnerabilities among the workers. A qualitative approach appeared to be the most appropriate method for exploration and obtaining in-depth understanding of the informal institutions and insights on how the institutions rendered venue workers vulnerable to HIV acquisition (32). Individual interviews enabled touching personal matters relating to informal institutions and related risk exposures (33). Through out the study we adhered to the guidelines for reporting qualitative research (34).

### Study setting

The study area (Greater Rakai) comprises two recently split districts of Kyotera and Rakai. According to the 2014 Uganda population census, greater Rakai had a population size of about 518,000 with a population growth rate of 2.06. The district comprises four counties, which are made up of 23 sub-counties, and three autonomous Town Councils—Kyotera, Lyantonde, and Rakai. The sub-counties comprise a total of 890 villages and trading centers (35). The main towns within Town Councils are Kyotera and Lyantonde. Other small urban areas are Kalisizo and Mutukula Trading Centre, and the district headquarters in Rakai town. The main economic activities in the district include agriculture and small business. Greater Rakai District was purposively selected into the study because of its high burden of HIV prevalence (12%) in Uganda (36), also highlighted in the recent PLACE 2018 survey (11).

### Recruitment

The study population consisted of workers and managers at social venues in greater Rakai district, Uganda. The young venue workers (under 25 years) and venue managers were recruited from 22 social venues across greater Rakai district. The sampling was performed using the purposive and convenient method. The study focused on young venue workers because literature shows that venue workers are typically young people. We purposively recruited workers who had worked at the venue for at least 2 months because of their familiarity with venue rules, processes, and procedures. The 22 study venues were identified from the priority list of venues with the highest HIV prevalence as per the PLACE 2018 survey in Rakai District (37). Primary data collection for this paper was delayed by COVID-19 and its restrictive preventive measures i.e., closure of social venues in Uganda. By the time of data collection, lock down restrictions were partially lifted and generally observation of the same was weak especially up country. Nonetheless, a few social venues (2) were not found operational at the time of data collection and were replaced with similar venues. We worked closely with local structures to approach the selected social venues for data collection. Table 1 shows the venues included in the study. At the selected venues, permission was first sought from the venue manager to include his/her venue in the study. Potential participants were then approached to discuss their eligibility for the study, study details, and willingness to participate. Those who were eligible and accepted to participate were recruited into the study. The eligibility guidelines for workers included, (a) worker at the venues, (b) sex both female and male, (c) age 15–24 years, (d) having worked at the venue for at least 2 months; and (e) willing to participate in the study. For the managers, the eligibility guidelines included having worked at the venue for at least 2 months; and willingness to participate in the study.

**Table 1:** Venues included in the study.

**Venue type**	**Venue size**				**Total**
	**Small**	**Medium**	**Big**	**Huge**	
Bar and lodges	1	5	2	–	8
Local brew serving establishments	2	1	–	–	3
Restaurants/pool tables sites	5	2	1	–	8
Hotels	–	–	1	2	3
Total	8	8	4	2	22

Venue size was determined by the number of customers at the venue during the peak hour (busy times). The small venues reported up to 30 customers during their busy time; medium venues reported between 30 and 80 customers, the big venues had between 80 and 150, and the huge venues had over 150 customers during their busy times. Relatedly, the young workers and venue managers were selected based on their age, position and or duration of work at the venue.

### Participants

At each selected venue, a manager and at least one young worker (15–24) were approached and consented for an interview. Priority was given to workers who had worked longest at the venue, because of their long experience and knowledge about the venue. We interviewed 44 young venue workers who had worked at the venue for at least 2 months. They were employed in different positions including waitresses (17), waiters (8), cleaners (4), security officers (6), pool table attendants (3), and workers of aside business at the venues i.e., mobile money services, butcher, and pedicure (6). We also conducted 22 in-depth interviews with venue managers, five of whom were also business owners. We never asked the workers and managers whether they identified as sex workers.

Supplementary Table A1 shows the characteristics of the study participants. Overall, 66 individuals participated in this study (44 workers, and 22 managers). Out of the 44 workers in this study; 31.8% (14) were males, and 68.2% (30) females. The age range was 15–24, with about 30% of workers under 20 years. The majority (91%) of the young workers were not married or living with a partner. More than half (56.8%) of the workers reported no or some primary education. Most (75%) of the young workers subscribed to Catholicism. Regarding the duration of working at the venue, about 70% had worked at the venue for not more than 1 year.

Regarding the managers, out of the 22 managers interviewed; 63.6% (14) were females, and 36.4% (08) males. The age range was 20–39, with about 27% of managers under 25 years. The majority (91.3%) of the managers were not married or living with a partner. Like the young workers, most (77.2%) managers subscribed to Catholicism. Contrary to the workers, more managers reported slightly higher education background; 59% reported secondary education. Regarding the duration of working at the venue, over 85% managers had worked at the venue for one or more years.

### Data collection

Two separate interview guides for in-depth interviews with young workers and venue managers were developed, respectively. The guides were translated into the local dialect (Luganda) and the interviews were conducted in Luganda. The themes covered in the guides included; recruitment and hiring—role profiles, person specification, knowledge, skills and experience; orientation and training; salaries, bonuses and other benefits; code of conduct—sexual harassment, dress code, substance use; working hours and breaks; health, safety and wellbeing; reporting and employee relations among others. Questions about the existing informal institutions were followed by probes to elicit further depth or breadth in the responses; as well as probes exploring details with respect to how particular institutions influenced the risk of HIV acquisition among the young workers. Some of the probing approaches used included; asking the interviewee to share more, silence, and the interviewer repeating the last point that the interviewee said to solicit for more (38).

The time allotted for in-depth interviews was 60–90 min but some interviews stretched to about 120 min because participants were allowed to step aside in case a customer needed a service. The interviews were held in a private spot at the venue, and at a convenient time identified by participants and there was flexibility to explore emerging themes. The interviews were audio recorded after obtaining consent from the participants and later transcribed verbatim. Following completion of the interview, each participant received Uganda shillings 10,000 ($2.7) compensation for their time.

In keeping with strong qualitative methodology, the research team debriefed daily after the target for the day was completed, and re-prioritized or revised inquiries in subsequent interviews as dictated by collected interviews (for example, emergent themes). Interviews for both categories of respondents continued until the team agreed that data saturation had been obtained on all topics (39). Prior to collecting the data, we held a 2-day training during which the study team reviewed the interview guides in detail, consenting process, field processes, and planned logistics for fieldwork. The research team comprised of the principal investigator, and four young research assistants below 30 years (two males and females) with graduate level of education, and research experience on topics related to HIV/AIDS, sexuality and/or alcohol use; as well as experience in conducting qualitative research studies. This was done to minimize age bias in reporting.

### Data analysis

This study used thematic content analysis technique that involves in-depth interpretation of the underlying meanings of the text and condensing data without losing its quality (40, 41). The IDIs were transcribed in Luganda and translated into English by a research assistant with experience in translation and transcription of qualitative data. The lead investigator read through all the transcripts several times while identifying the emerging themes. A codebook was developed using deductive codes based on the themes of Rhodes and Simic's model of HIV risk environment (29). Both inductive and deductive coding approaches were applied (39). The lead investigator coded the transcripts using Atlas ti (version 22). The research team reviewed the coded text to generate themes and identified illustrative quotations for each theme (42). The analysis was discussed among the research team members and discrepancies on coding and other issues that required clarity were settled by discussion. Quotes that best described the various categories and expressed what was aired frequently in several groups were chosen.

To enhance credibility, we selected participants from different types of venues, different staff positions, gender, and age sub-groups. Data was collected until saturation was reached which allowed identification of all relevant aspects to answer the research question. Preliminary results were shared to participants for input and elaboration. To assess dependability, peer checking by another team member to re-analyze some of the data was done. Team members also discussed discrepancies on coding and other issues that required clarity. Data collection and analysis has been described in-depth to allow replicability.

### Ethical considerations

This study received approval from the Research Ethics Committee at Ghent University, Belgium (number: 2019/0593); Higher Degrees Research Ethics Committee at the Makerere University School of Public Health (number: 868) and Uganda National Council of Science and Technology (number: HS1536ES). Permission was sought from relevant offices at national and sub-national levels in Uganda including district health offices, venues authorities etc. Informed consent was obtained from study respondents. Confidentiality and privacy were upheld during all phases of the study.

## Results

**Figure 1:**
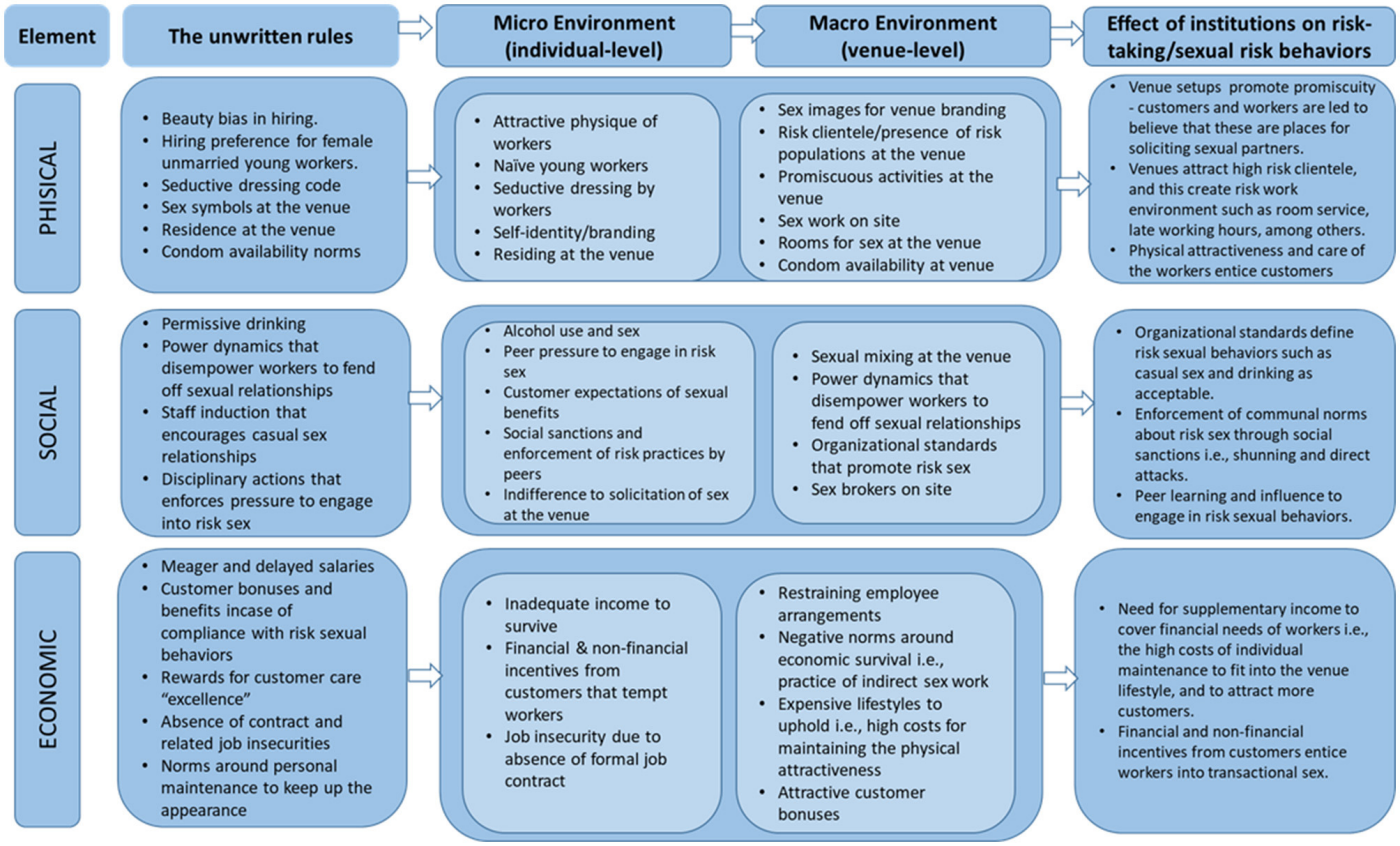
Informal institutions and their effect on risk-taking/sexual risk behaviors.

Figure 1 summarizes the key results on the informal institutions at the venues, and the attributes through which these institutions influence vulnerability for HIV for venue workers. The findings are organized into three broad themes based on Rhodes and Simic's model of HIV risk environment: physical, social, and economic related informal institutions (27). The fourth theme of the HIV risk environment framework (policy) has been dropped because it is implied in institutions. The influence of informal institutions on HIV vulnerability was derived from participants responses. This was done by synthesizing the risk exposure opportunities arising from the reported informal institutions. No major differences were observed in the informal institutions or rules across venues. Where differences were observed, they have been reported.

Overall, the institutions at the venues were a mix of official and social rules i.e., some institutions were created, communicated, and enforced within officially sanctioned channels i.e., communicated by individuals with positions of authority such as venue supervisors or managers. On the other hand, other institutions were created, communicated, and enforced outside of officially sanctioned channels. Respondents mentioned that the latter category of institutions were verbally communicated by peers who had worked longer at the venues. The institutions broadly influenced HIV vulnerability by being sexual agencies. For detailed description of institutions at social venues, see Supplementary Table A2. Regarding communication of the official institutions, one venue manager remarked.

“*Depending on what someone [worker]has been hired to do. [….] if it is a waitress, I will verbally tell her what is expected of her; sweeping, cleaning, arranging the chairs and also explain to her, her relationship with the customers… for me, the customer is always right*.”, **31-year-old female manager at a guesthouse/lodge**.

### Theme 1: Institutions that are physical or observable

This theme reflected content related to institutions of a physical nature or institutions that can be observed or institutions with physical elements. These included visible institutions at micro (individual) level as well as those at macro (venue) level.

*Visible or observable institutions at micro level*: The most common visible institutions at individual level that were unanimously described by study participants included physical conduct norms, and workers' residence at the venue. Study participants reported physical conduct of a sexual nature such as consensual and non-consensual sexual conduct. Participants described non-consensual sexual activities as conducts of a sexual nature (both verbal and physical) that occurred without the workers' consent. They mentioned several forms of non-consensual sexual behaviors including uninvited sexual or suggestive comments or jokes about the workers' physical looks; sexual gestures through hand or body movements; requests for sexual favors, inappropriate physical touching of the workers' body parts including, fondling, hugging or kissing, sexual intercourse, discussions about sexual relations/fantasies at work among others. Another physical institution mentioned was that some workers mostly females were residing at the venue. Regarding sexual gestures, one participant remarked:

“*……..at night, the lights are dim, the music is loud and the venue is very busy. Most of our customers drink and as they get tipsy, you expect them to touch you, make sex comments, whether you like it or not because they're not sober [drunk]*”. **24-year-old female venue worker_ waitress at a bar and lodge**.

*Visible or observable institutions at macro level*: The most common visible institutions at venue level that were described by study participants included beauty bias in hiring, hiring preference for female unmarried young workers, seductive dressing code, presence of provisions for sex on site, condom availability norms, and the sex symbols at the venue. Both managers and workers explicitly described hiring biases at the venues. These were mainly created and implemented by the hiring authority i.e., supervisors, managers, and venue owners. The most common selective hiring norms reported included preferences for young workers below 30 years, female workers for roles interfacing with clients, workers with attractive physique, and workers not in committed relationships i.e., the unmarried, divorced or separated individuals. Regarding beauty bias in hiring, and seductive dressing code, some participants remarked.

“*I don't employ married people..., because their husbands may fight when they find them here seated with male customers. Male customers often come here just to relax, at times they buy drinks for the workers and in turn the girl [worker] may get drunk. In case they were married, their partners would perceive it negatively*”, **27-year-old female manager at a Bar and Lodge**.

“*Even if someone is old but good-looking, they would be favored..., as you know, men just love taking alcohol in company of beautiful ladies, so regarding appearance, the person looking for a job here [at the social venue] must be physically eye-catching*”, **23-year-old male worker_ Security guard at a Bar and Lodge**.

“….*no one tells you but to fit in, you must maintain an attractive physical appearance. Especially the way you [worker] dress and make up. Normally, we put on short and tight stylish clothes, and extremely care for our skins to match with the lifestyle at the venue and remain attractive*”, **21-year-old female worker_ waitress at a restaurant**.

Participants also mentioned that some venues had provisions for having sex. These were mainly accommodation rooms at lodges, hotels or just spare rooms used in case of need. Relatedly, in some venues especially those with provisions of having sex on site, condoms were occasionally available in condom dispensers, in the rooms or at the counter and provided on request. Furthermore, participants also mentioned venue branding norms such as dim lighting, promiscuous activities like stripping, movies and music, dark corners, and erotic paintings as visible informal institutions at the venues.

### The influence of physical-related (observable) institutions on HIV vulnerability

Institutions such as selective hiring, seductive dressing code, and residing at the venue affected the physical venue environment by creating set-ups that promoted promiscuity, attracted risk groups like sex solicitors, and drew attractive workers that enticed the customers into sexual risk behaviors i.e., drinking, casual sex, condomless sex, and sex work. Respondents explained that the seductive dressing code, and selective hiring at the venues attracted mainly naïve and physically attractive young workers, which stirred customer's expectations of sexual benefits. Related, naïve young workers were less likely to negotiate for safer sexual relationships and condom use. Respondents also mentioned that the skimpy dressing attracted sexual harassment acts including forced sex. Managers mentioned that some workers physically conducted themselves in a manner that portrayed them as solicitations of sex, hence drawing customers' sexual advances. Residing at the venue was unanimously described as risk factor for engagement into sex by respondents. They mentioned that it increased chances of having sex on site. One participant remarked.

“*All of us the female workers reside here at the venue. We were given a room by our boss where we sleep… often times we do not sleep in that room because customers ask us to spend a night with them [sex] and they give us money. The pay is usually high depending on one's negotiation powers, and whether or not you are using a condom. ….when we get such a customer, we sleep in the other wing [the lodges].”*, **20-year-old female worker_Waitress at a Bar and Lodge**.

Another participant shared her experience of risk exposure triggered by room service:

“*The guy [customer] came here and…… chose me to serve him* […….] *in his hired room… While serving them [customer and friends] he told me to join them for a conversation, which I did because it is part of customer care….., he then asked for sex…, I told him he couldn't have sex with me because of the two bottles of soda he had bought for me. He wanted to add me Uganda shillings 5000* [USD 1.3]*, but when I refused it turned into a fight. I tried to escape but, he hit me with a bottle here on the head…”*, **22-year-old female worker_Waitress at a Bar and Lodge**.

Relatedly, respondents reported that presence of rooms at the venues meant that these rooms could be hired by customers for relaxing or having sex. It was reported to be easy for a customer hiring a room at the venue, to solicit sex from workers. Some respondents mentioned that customers visited the venues in hopes of finding a sexual partner, and workers were part of the group to draw from. Respondents also believed that most physical venue features influenced workers and customers' perceptions of the venues and created a conducive physical environment for sexual relationships. One respondent remarked.

“*You* [worker] *are not allowed* [by venue managers] *to sleep outside* [away from the venue]. F*or example, the girls* [female workers] *who get guys from here* [venue] *are not allowed to go with them outside because we have lodges here, they use the rooms here at the venue*”, **22-year-old female manager a Bar, Restaurant and Lodge**.

### Theme 2: Social institutions at the venues

The social institutions evolved around social interactions between peers, managers, and the customers at the venues. These included social rules at micro or individual level as well as those at macro or venue level.

*Informal social institutions at micro (individual) level*: The most common social institutions at individual level that were reported by study participants included permissive drinking, customer expectations of sexual benefits, social sanctions, and enforcement of risk practices by peers, indifference to solicitation of sex at the venue, power imbalances, and staff induction that encourages casual sex relationships. Drinking alcohol, and sexual relationships at the venues were acceptable. However, excessive drinking, and committed sexual relationships were disapproved. Respondents highlighted that worker-to-worker sexual relationships were socially unpopular, and male workers engaging in transactional sex at the venue was less common. Workers considered their peers at the venue as family and knew each other's risk sexual practices, which discouraged relating sexually. According to the participants, “inappropriate” behaviors were punishable either socially and or formally by managers or venue owners. Regarding peer influence, some participants remarked.

“*When I had just started working at this venue, I became close friends with one worker* [name withheld*] who had worked here for about three months. She helped me to understand how things are done here [venue]. For example, I didn't know how to manage a customer's sexual advances but she* [fellow worker] *supported me until I got confident. Now I can face off a customer in case I am not interested or negotiate better*”, **21-year-old female worker_Waitress at a Bar and lodge**.

“*At the beginning there is always that fear or conscience that keeps reminding you that this or that* [practices at the venue] *is wrong. But with time and as you see your peers [fellow venue workers] doing the same or not minding it, you get comfortable and start engaging in things like, accepting customer sexual requests or even transactional sex*”, **20-year-old female worker_ Waitress at a Bar**.

*Social institutions at macro or venue level*: The most common social institutions at venue level reported by study participants included power dynamics that disempower workers to fend off sexual relationships, norms about sexual mixing at the venue, organizational standards that promote risk sex, and presence of sex brokers on site. Participants universally emphasized the rights and powers of customers at the venues. They explained that customers were highly regarded at venues and if they complained about a worker, they would be listened to by management. One respondent remarked.

“*We are here to give very good services to our customers, but should a customer try to seduce you, you still remain calm….. if you are rude with the customer, he may not come back*”, 22**-year-old female worker_Waitress at a bar and restaurant**.

Another participant said:

“*…. should the customer report you to the manager, you risk losing your job. That is why it is better you serve them with care*”, **24-year-old male worker_Waiter at a hotel**.

Discussions further revealed rigid reporting structures at almost all venues which highlighted power dynamics between workers and management. A highly centralized “command and control” style was reported at most venues. All workers and customers reported to the managers. The managers had all powers to hire and fire including immediate dismissal in cases of customer related grievances. Relatedly, respondents reported that contracting at the venues was verbal, except for the huge venues. The contracts were outlined and agreed to via spoken communication between the worker and the manager.

### The influence of social institutions on HIV vulnerable

Institutions around social interactions at the venues influenced vulnerability to HIV by defining sexual risk behaviors such as drinking, casual sex as normal or acceptable. For example, respondents unanimously mentioned that peers played a critical role in influencing their sexual behaviors at the venue. They promoted shared values on casual sex or sexual relationships formation at the venue. According to some workers, peer pressure to engage into sex with benefits made it lighter for them to initiate and engage in similar risk behaviors. In addition, peers enforced communal norms around sex and condom use through social sanctions such as shunning and direct attacks. Most of the workers indicated that the longer they stayed at the venue, the more confidence they gained at discussing and engaging in sexual relationships at the venue.

Relatedly, almost all respondents including managers reported that customers came in hopes of buying sex or initiating sexual relations at the venue, and this positioned these venues as avenues for sexual relationship initiation and harassments. Respondents also reported that the customer care (special treatment) expected of them, to some extent raised customer expectations for sexual benefits.

Furthermore, respondents alluded to the policing practices at the venues i.e., reprimands, suspension, and instant dismissals by managers as catalysts for risky sexual behaviors at the venues. Reprimands, suspension, and instant dismissals were mainly related to customer mishandling. Moreover, most customer complaints were due to the failure by the workers to meet customer expectations including sexual benefits. Study participants mentioned that the policing practices at the venues re-enforced negative organizational standards or values such as casual sex, and disempowered workers to fend off sexual risk behaviors. One venue worker mentioned:

“*Good customer care is what matters the most here* [at the venue]. *If you want to keep your job, please the customers or else the manager will tell you off or even dismiss you. Otherwise, one* [a worker] *must put up with a lot of customer shit* [wrongdoings]”, 24-year-old female worker_ Waitress at a Bar and Lodge.

Sometimes, workers were given talks on how to manage customer relations. One venue manager remarked:

“*We don't punish them* [worker] *rather we talk to them on how to manage relationships. You teach her* [worker] *about how things are done here* [……...]”, **28-year-old female manager at Bar and Lodge**.

At venue-level, social institutions such as negative organizational values or standards promoted casual sexual relationships, promiscuous acts i.e., sex themed talks, gestures that portrayed sex-related signals at the venues. Relatedly, respondents reported sharing stories and past experiences regarding how to survive at the venues. Some respondents reported that some of the stories shared offered wrong perspectives on sex and relationships and discouraged individual protective values among the new workers. Some venues were reported to have sex brokers; peers who helped others to initiate and negotiate for sex with benefits.

The workers and managers also reported drinking norms as a condition that promoted engagement into sex at the venue through impairing one's decision-making ability. Managers and workers, shared experiences of peers unknowingly having condomless sex with customers when drunk.

“*We are allowed to drink [alcohol] but warned to be aware of our limits. If I know my limit is two bottles of beer, I will not do four beers because it can lead me to doing things [sex] unconsciously…”*, **30-year-old female manager at a bar and restaurant**.

### Theme 3: Economic institutions at the venues

This theme reflected content related to informal institutions of economic nature or institutions concerning resource generation, distribution, and utilization at the venue. These included economic related institutions at micro (individual) level as well as institutions at macro (venue) level.

*Economic institution at micro (individual) level*: The most common economic related institutions at individual level that were described by study participants included norms around personal maintenance to keep up the appearance and to manage within the meager wages. Venue workers openly mentioned that they engaged into risk sexual behaviors at the venue because of the need to supplement the meager and delayed wages in order to meet their requirements i.e., maintaining their lifestyle at the venue, upkeep (food, clothing, healthcare) as well as taking care of their dependents. One respondent reported:

“*I have many requirements that need money… I need to buy nice clothes, make-up, Vaseline, food but also, I have a child to take care of back home. I use part of my salary to take care of my needs, but it is never enough and sometimes it delays….I found other means to survive…. If a customer offers a pay for sex, sometimes I allow and usually the pay for condomless sex is very attractive……*”, **23-year-old female worker_Waitress at a Bar and lodge**.

*Economic-related institutions at macro (venue) level*: The most common economic related institutions at venue level that were reported by study participants included meager and delayed salaries, customer bonuses and benefits in case of compliance with risk sexual behaviors, rewards for customer care “excellence”, and norms around personal maintenance to keep up the appearance. Regarding wages, customer cash tips and bonuses, one respondent mentioned:

“*I am paid UGX 3000* [$0.83] *per day but every day I go home with a minimum of UGX 10000* [$3]. *On a good day, I can even go home with about UGX 35,000 [$10]. It really depends on the customer cash tips received and if a worker is willing to offer additional services [sexual], they even earn more*”, **19-year-old female worker_Waitress at a Local brew serving establishment**.

### The influence of economic institutions on HIV vulnerability

Inadequate income, attractive financial and non-financial benefits from customers, and expensive lifestyle, were the three economic related institutions commonly reported by the study participants. Almost all study participants mentioned the three institutions as a major reason for engaging in sexual risk behaviors at the venues. They explained that workers engaged in sexual risk behaviors in order to supplement the meager salaries. The supplementary income was reported to assist in maintaining an expensive lifestyle at the venue, which included buying cosmetics and clothes to maintain an attractive physical appearance. Respondents also emphasized that the attractive financial and non-financial benefits from customers such as cash tips, gifts, and high offers for sex, enticed them to give in including unprotected sex. One participant remarked.

“*Our boss doesn't give us everything…., you may also have other financial needs back home, especially workers with children* [……...] *you end up sleeping* [having sex] *with this man* [customer] *for extra money. That way you can use this* [payment for sex] *UGX 20,000 and send UGX 10,000 back home, UGX 8000 for Vaseline and use UGX 2,000 for breakfast. Then you can save the salary*”, **21-year-old female Waitress at a hotel**.

At venue level, participants mentioned gaps in salary payment arrangements such as small irregular wages that forced workers to practice transactional sex for economic survival. They also highlighted job insecurity caused by absence of job contract, and norms around economic survival through transactional sex at the venue. Other respondents believed that the financial constrains discouraged individual positive or protective values i.e., abstinence from sex, and condom use.

## Discussion

This qualitative study explored the informal institutions or unwritten rules at social venues, and how they influenced HIV vulnerabilities among venue workers in Uganda. The robustness of our results was strengthened by using an HIV risk environment conceptual framework by Rhodes and Simic (29) for researching multiple environmental factors that produce health risk and vulnerability. The study found several informal institutions that circulated within the venues that are tied to various behaviors that may put workers at risk, such as alcohol use, power imbalance, financial pressure to have transactional sex with customers, indifference to solicitation of sex at venue, and a lack of employment security at the venues among others. These institutions were both officially and socially created, communicated, and sanctioned. Our findings on the informal institutions at social venues coincide with findings from earlier studies conducted in Asia, that reported venue attributes indicative of institutions at social venues (9, 20). A study by Hsu and colleagues reported that entertainment workers in Cambodia were typically employed informally by their employers, and were subjected to long working hours, sexual harassment, and violence. In addition, many workers who sold beverages were forced into excessive alcohol consumption as part of their work and others were expected by their employers and clients to provide sexual services (20).

In terms of HIV vulnerability, the study found that informal institution at social venues shaped a risk environment at the venues by creating risk exposure opportunities, conditions that initiated sex i.e., sexual violence and coercion or influenced workers to engage into sexual risk behaviors such as low condom use, reduced opportunity to discuss HIV status before sex, multiple sexual partnerships, low negotiation opportunity among others. Our analysis reveals a convergence of several risk exposure opportunities within the venue environment (Figure 1) that were shaped by the informal institutions at the venues. These included among others, physically attractive workers, venue brand and clientele, sex work on site, sexual violence and coercion, shared values on sexual relationships, social suctions, organizational venues, culture, and symbols as well as meager and delayed salaries. Most of these risk exposure opportunities in this study were alluded to literature, including in an international handbook of Gender and Poverty by Chant (43). The book highlighted the pressures faced by bar girls and others in the entertainment sector regarding engaging in sexual relationships at venues for economic reasons (43). This highlights the need for venue-centered programs and policies that embrace the venue context and promote collaboration with venue actors in implementing interventions that address the social structural factors that shape and determine the risks at venue work environment. Moreover, the case for venue-based HIV prevention interventions has been demonstrated in different setting in Africa (16, 44, 45). Venue based HIV prevention interventions should embrace a social norms approach in order to address the institutions at the venues that guide or constrain social behaviors of individuals at the social venues.

Relatedly, an earlier study reported that male clientele at social venues in Nigeria commonly battered playfully with female workers and in most cases these encounters evolved into sexual relationships (46). It was also highlighted that the line between sex work and tavern work was blurry since often times the relationships initiated at the venues involved transactional practices although they were not viewed as commercial sex by the parties involved (46). This is also similar to earlier PLACE studies in Uganda and Tanzania, that reported transactional sex practices among workers at social venues (11, 23, 30, 37). Studies in Asia have also reported high prevalence of both commercial sex work and casual sex behaviors in women working in entertainment establishments (17, 18, 47). These studies highlighted that while many of the services offered at social venues are non-sexual in nature, some female venue workers in certain establishments offered additional sexual services to customers after or in lieu of standard services i.e., alcohol. Workers of social venues have also been reported to engage into sex work with their male patrons to supplement their income (13, 21). These findings allude to poverty and the fundamental role that financial insecurity plays in HIV vulnerability. In this study, venue workers explained the toll of financial hardships in their lives, and families, and the extent to which they would go to supplement their salaries/wages in order to care for themselves, children, and families.

In this study, alcohol consumption, a known risk factor for HIV was reported. Workers at almost all venues mentioned that they were allowed to drink alcohol, including drinking with their customers. Allowing workers to drink could be partly to entice customers to buy more drinks, in addition to facilitating flirtatiousness between workers and customers. Our finding coincide with two studies conducted in Cambodia among Female Entertainment Workers (FEWs), that reported drinking among FEWs, and specifically due to pressure from clients and supervisors (20, 48, 49). Another study estimated that 62% of FEWs drink alcohol every day at work and about half of those who drink daily (49.9%) reported drinking more than 55 units in a week (cans or bottles for beers and glasses for wines and heavy alcoholic drinks) (48). Notably, literature highlights that alcohol elevates HIV vulnerability though multiple mechanisms related to decision-making, including dampening protective cues, altering perceived expectations regarding sexual expectancies, and reducing sexual inhibitions, thus facilitating the likelihood of risky sexual behavior (50, 51).

Lastly, many of the practices reported in this study such as sexual assault, rape, alcohol-related non-consensual sex, partner violence, may constitute sexual violence, a known risk factor for HIV transmission (52), and would require immediate response, however this study took a different perspective. It documented these practices and the other work that follows this paper shall involve engaging the institutions mandated to address these practices. The broader research work in which this paper is arched has arrangements to dialogue with the policy makers in Uganda on this broader phenomenon. Notably, such practices have also been reported elsewhere among venue workers. For example, a study among high-end entertainment center workers in Hunan Province, China reported that employees at these centers were often young, female rural-to-urban migrants and vulnerable to sexual violence and exploitation (53). Moreover, in this study, workers who reported sexual violence related practices did not refer to it as so. This further highlights the power dynamics between the workers, customers, and the supervisors.

### Strengths and limitations

This paper contributes to an emerging literature that highlights the importance of social and structural factors in shaping HIV risk by bringing into focus one element that has been hidden in these debates—the role of institutions (in this case informal institutions) in structuring risk environments and ultimately influencing vulnerability to HIV among individuals working in social environments. The paper highlights the informal institutions that interact to shape the venue risk environment, which in turn influences or promotes the risk of HIV transmission at social venues. The strengths of the paper lie in the use of qualitative data, which allow for a better understanding of the mechanisms that may lead to risky sexual behaviors among venue workers, and thus higher rates of HIV/AIDS.

We used multiple data sources i.e., venue workers, and managers to develop a comprehensive understanding of the phenomena hence data source triangulation.

Important to note are the limitations of this study. The focus of the current study was the venue. We explored the informal institutions or the unwritten rules at the venues and not any other structural factors outside the venue i.e., existing laws governing these places, yet these may also influence the venue risk environment. External structural factors such as economics of the broader hospitality industry, public policies related to venue work environments, should also be explored in future research. Furthermore, participants were interviewed at their place of work could have introduced bias in a truthful account of their workplace in fear of retaliation. However, it was minimized by conducting the interviews at a private spot within the venue. Despite the limitation, we believe the study presents important insights into how informal institutions at social venues shape the risk work environment and subsequently HIV vulnerability, hence informative for venue-based HIV prevention programs.

## Conclusion

Findings from this qualitative study give an overview of how the informal institutions at social venues create HIV vulnerably among venue workers in Uganda. The study found that informal institutions at social venues were both officially and socially created, communicated, and sanctioned. We found that the informal institutions at social venues shaped a risk environment at the venues by creating risk exposure opportunities within the venue environment. This in turn-initiated sex, and or influenced engagement into sexual risk behaviors by workers. Therefore, emphasis should be put on modifying the negative institutions at the venues that shape the risk in venue environment. This could be done through venue-centered policies and programs that consider the venue social context, engage, involve, strengthen, and support venue actors who are central at naturing venue institutions. Venue-based HIV programs should integrate social norms interventions to influence venue workers' behaviors. Venue actors such as managers should also be involved in enacting and enforcing venue-based policies that regulate negative unwritten institutions at the venues. Provision of HIV protective measures, and venue-based capacity building programs for workers should also be enhanced to empower venue workers to navigate the existing risk exposures at the venues. Examples of policies that could regulate negative institutions, include; venue licensing requirements or standards on staff contracting, minimum wage, operating hours, and condom availability at venues at all times.

## Data availability statement

The raw data supporting the conclusions of this article will be made available by the authors, without undue reservation.

## Ethics statement

The studies involving humans were approved by IRB Ghent University, Makerere University School of Public Health IRB, Uganda National Council for Science and Technology. The studies were conducted in accordance with the local legislation and institutional requirements. The participants provided their written informed consent to participate in this study.

## Author contributions

SB: Conceptualization, Formal analysis, Investigation, Methodology, Project administration, Validation, Writing—original draft, Writing—review & editing. KM: Conceptualization, Methodology, Supervision, Writing—review & editing. FS: Conceptualization, Methodology, Supervision, Writing—review & editing.
