# Supplementary material for: The unwritten rules and HIV: a qualitative study of informal institutions and HIV vulnerability among workers at social venues in Uganda
Source: Front Public Health. 2023 Dec 14;11:1288058. doi: 10.3389/fpubh.2023.1288058 (PMC10752962; doi:10.3389/fpubh.2023.1288058)
Supplement: Supplementary file 1 [file Table_1.docx]

**Table A1: Description of study participants by their socio-demographic characteristics**

| Venue Characteristics | Total sample of workers  (n=44) | Young workers | | Total sample of managers  (n=22) | Venue managers | |
| --- | --- | --- | --- | --- | --- | --- |
|  |  | **Female**  **(n=30)** | **Male**  **(n=14)** |  | **Female**  **(n=14)** | **Male**  **(n=8)** |
| Age category |  |  |  |  |  |  |
| 15-19 | 13 (29.5%) | 08 | 05 | 00 (0% ) | 00 | 00 |
| 20-24 | 31 (70.4%) | 22 | 09 | 06 (27.2%) | 06 | 00 |
| 25+ | 00 (0%) | 00 | 00 | 16 (72.7%) | 08 | 08 |
| Marital status |  |  |  |  |  |  |
| Currently married | 04 (9%) | 02 | 02 | 04 (18.2%) | 3 | 01 |
| Previously married/Separated | 05 (11.3%) | 05 | 00 | 01 (4.5%) | 01 | 00 |
| Single/not living with a partner | 35 (79.5%) | 23 | 12 | 17 (77.2%) | 10 | 07 |
| Level of education |  |  |  |  |  |  |
| No education | 08 (18.1%) | 05 | 03 | 00 (0%) | 00 | 00 |
| Primary not completed | 17 (39%) | 11 | 06 | 03 (13.6%) | 03 | 00 |
| Primary completed | 11 (25%) | 08 | 03 | 06 (27.2%) | 04 | 02 |
| Secondary not completed | 07 (16%) | 06 | 01 | 11 (50%) | 05 | 06 |
| Secondary completed | 01 (2.3%) | 00 | 01 | 02 (9%) | 01 | 01 |
| No of years working at venue |  |  |  |  |  |  |
| Less than one year | 70.4% (31) | 25 | 06 | 03 (13.6%) | 02 | 01 |
| 1-3 years | 27.2% (12) | 05 | 07 | 14 (63.6%) | 09 | 05 |
| 3+ years | 01 (2.3%) | 00 | 01 | 05 (22.7%) | 03 | 02 |
| Religion |  |  |  |  |  |  |
| Islam | 04 (9%) | 02 | 02 | 01 (4.5%) | 01 | 00 |
| Catholic | 33 (75%) | 23 | 10 | 17 (77.2%) | 11 | 06 |
| Protestant | 07 (16%) | 05 | 02 | 04 (18.2%) | 02 | 02 |
