# Supplementary material for: The unwritten rules and HIV: a qualitative study of informal institutions and HIV vulnerability among workers at social venues in Uganda
Source: Front Public Health. 2023 Dec 14;11:1288058. doi: 10.3389/fpubh.2023.1288058 (PMC10752962; doi:10.3389/fpubh.2023.1288058)
Supplement: Supplementary file 2 [file Table_2.docx]

**Table A2: Overview of the informal institutions or unwritten rules at socials**

| Category | Subcategory | | Explanation |  |
| --- | --- | --- | --- | --- |
| Attracting candidates | Solicited applications | | Job announcements were used by big and huge venues like hotels. |  |
|  | Unsolicited application | | Main approaches for worker attraction at small venues were walk-ins, and referrals.  Owners of small venues commonly worked at venues. |  |
| Role profile | Role-sex preference | | Female workers were preferred for customer interface roles i.e. serving.  “..*male workers often do back door roles like cooking*” |  |
|  | Duties and responsibilities | | The overall responsibility of all workers was customer satisfaction.  “….*serving with a smile is must as well as good customer care*”.  Common duties and responsibilities at the venues: venue or department management, serving customers (including room service), keeping safety and security, cooking, balancing books of accounts, and cleaning. |  |
|  | Reporting relationships | | A vertical reporting structure was common at all venues, with a single individual [manager or venue owner] at helm of the venue.  All workers and customers reported to the managers, except for some big and the huge venues that had departmental managers. |  |
| Person specification | Working age | | 18-30 years olds were preferred and believed to be naive and attractive.  Under 18 years but looking physically mature were employed.  Local brew bars did not have age bias in hiring. |  |
|  | Physical appearance | | Physical attractiveness was highly considered especially for customer interface roles.  Beautiful, light skinned, stylish, and attractive physique were among the desired features of a worker. |  |
|  | Other socio-demographics | | Female workers were more preferred than males.  Unmarried or divorced workers were desired, and believed to easily fit in.  Religion and education background were not considered in hiring, accept for some roles that required some reading and writing ability.  Workers with children were disliked. |  |
| Experience, Knowledge and skills | Experience | | Previous experience was not considered in hiring, accept for the huge venues.  “..*I hire* *anyone provided they are willing to abide by the rules here”.* |  |
|  | Knowledge | | Readiness to work, and willingness to offer good customer care was emphasized.  Specifically, willingness to tolerate customers, work late hours & general good conduct. |  |
|  | Skills | | Skills were needed for only specific roles such as cooks. |  |
| Staff contracting | Employment contracting process | | Verbal and open-ended contracts were common especially at small and medium venues.  Managers orally explained the dos & don’ts at the beginning. |  |
| Salaries, bonuses and benefits | Salaries | Remuneration ranged from Uganda shillings 2,000 to 5,000 per day.  Cash payments were common.  In-kind payments (beers) and commissions were highlighted in a few medium venues. Common payment schedules included: daily, weekly, and monthly at medium and huge venues. Salary delays were commonly reported. | | |
|  | Bonuses | Cash bonuses were common in cases of exemplary customer care and or extra services (sex) to customers.  Other bonuses included; good meals and parties in cases of good performance. | | |
|  | Benefits | Common benefits included; provision of accommodation and meals to workers.  Cash tips from customers were reported at all venues.  Accommodation was mainly provided to female workers, and sex workers.  Partial meals were provided and workers expected to supplement. | | |
| Performance and Reward | Performance indicators | Major performance indicators included; good customer care, and employee conduct. In some venues, good performance meant serving many customers. | | |
|  | Performance monitoring | Performance monitoring was primarily done by the managers. | | |
|  | Reward | Good performance was rewarded by cash bonuses from customers and in a few places, managers. Customers also offered in-kind gifts for special treatment. | | |
| Employee relations | Employee-employer relations | “*I have not heard of any worker-manager relationship here* [ venue] *but even still, if it did happen, they are both adults*”. | | |
|  | Employee-customer relations | Open relationships were promoted at the venues.  Employees were not allowed to have a main partner and in case of a main partner, partners were not allowed to visit the venue.  Customer-worker and worker-manager relationships were allowed.  Some venues had brokers to help people find a sexual partner. | | |
|  | Employee-employee relations | “*We* [workers] *consider each other brothers and sisters. Besides it is hard to fall for each other because we are aware of each other’s sexual relationships*”. | | |
| Code of conduct | Work schedules | Long and night working hours were common.  Working time highly depended on customer availability.  Working hours ranged from 7.00 am until when the last customer left the venue.  Long leaves of absence were uncommon. Workers were given a day or half day offs for individual general cleaning and these were not paid. | | |
|  | Dressing code | Short, tight and stylish dressing was promoted.  A few venues had uniform but also tight or short.  Attractive dressing was particularly encouraged in the evening hours especially at drinking venues. | | |
|  | Substance use | *“We* [workers] *are allowed to drink alcohol…, even to drink with customers as we give them company. But it is not allowed to use drugs or get drunk at work”.* | | |
|  | Customer powers | Customer is the boss, was a common rule across all venues.  Customers had excessive rights and powers over the workers. | | |
| Health, safety and well-being | Health, safety and wellbeing | Condom availability at the venue, and advise from managers to play safe were the health aspects reported.  In some venues, workers were given treatment or first aid in case of sickness or emergency while at venue.  Fights and grudges were generally condemned.  Internal measures for mediating in cases of conflict included., hearing, counselling, rebuking and dismissal in cases of customer grievances. | | |
| Disciplinary system | Sanction approaches | Reprimands, suspension and termination were the common sanctions at the venue. Social sanctions such as shunning, quarreling, and fights were also highlighted. Customers were also suspended from some venues in cases of grave misconduct. | | |
|  | Positive approaches | “*We have a good boss. He often talks to us on how to “play safe”. He also gives us bonuses and benefits like good meals in case of good performance*” | | |
| Culture | Perceptions | Overall, there was a normative perception of risky sexual behaviors among workers. | | |
|  | Rituals | There were many informal procedures at the venues i.e., peer to peer induction, open discussion of sexual relationships, discussing customers, transactional sex practices etc. | | |
|  | Cultural cues and clues | Respondents reported universal beliefs, values, language and social norms about sex, sex work, and accepted behaviors in general i.e. vulgar talks were common.  Venue culture easy passed from one regime of workers to another through sharing of previous experiences at the venues, and stories surrounding the venue. | | |
| Off-boarding | Voluntary termination | The common formal off-boarding practices across the different types of venues included: employer advance warning, employee verbal notice, and employee quit. | | |
|  | Involuntary termination | Salary withdraws, dismissal, and layoffs were the main types of employee off-boarding practices at the venues. | | |
|  | Termination processes | Notice of termination were barely present at any venues.  Common reasons for dismissals were employee misconduct especially towards the customer and manager.  No compensations were given upon termination. | | |
| Others | Symbols of the venue | Rules around the visible organizational structures and processes like organization of services, deem lighting, displayed pictures. | | |
